# A hands-on approach to personalizing palliative care: the role of osteopathic manipulative treatment

**DOI:** 10.3389/fmed.2026.1894142

**Published:** 2026-07-01

**Authors:** Megan Bryant, Mikayla Dennison, Sharon Kim

**Affiliations:** Clinical Center (NIH), Bethesda, MD, United States

**Keywords:** non-pharmacologic analgesic techniques, osteopathic manipulation (osteopathic manipulative treatment/osteopathic manual medicine), pain management, palliative care, serious illness, treatment modalities

## Abstract

Osteopathic manipulative treatment (OMT) is an integrative modality that has proven benefits for patients suffering from physical and psychological ailments. Though the impact of OMT has been preliminarily studied in various patient populations, research and implementation of this treatment option has been limited. Within palliative medicine, integrating OMT into treatment plans can help to address both the physical and non-physical burdens of serious illness.

## Introduction

Precision medicine is centered on the individualization of care based on multiple factors. Osteopathic manipulative treatment (OMT), also known as osteopathic manipulative medicine, is a personalized therapeutic intervention, and therefore an ideal opportunity for improving patient care within precision medicine. OMT is often considered an adjunctive therapeutic modality that can be used to address common complaints such as musculoskeletal pain, though the utility of therapeutic touch and manipulative intervention has the potential to provide additional relief from a myriad of physical symptoms such as headaches, nausea, constipation, peripheral edema, and shortness of breath in addition to psychological symptoms such as depression and fatigue.

More specifically, OMT targets somatic dysfunction, defined as “impaired or altered function of related components of the somatic (body framework) system—skeletal, arthrodial, and myofascial structures, and related vascular, lymphatic, and neural elements” (1). Clinicians assess and diagnose these dysfunctions using a structural examination, then treat them to optimize function, improve pain, and restore homeostasis. Treatment techniques involve manipulation of the affected or surrounding tissue. Because the structural examination, diagnosis, and subsequent treatment plan are uniquely tailored to each patient’s presentation, OMT embodies the foundational principle of precision medicine.

Palliative medicine is a specialty within which providers work to improve quality of life by managing the physical and non-physical burdens of serious illness. The integration of OMT to decrease symptom burden naturally expands the range of non-pharmacologic treatment options available to palliative care providers. A nationwide survey of palliative care providers in the United States found that providers recommended complementary and integrative medicine (CIM) an average of 6.82 times per month, with mind–body medicines, massage, and acupuncture/acupressure as the most commonly recommended modalities—though providers recommended CIM less frequently than patients reported using such therapies, suggesting a gap between patient interest and provider recommendation (2). OMT represents an underutilized modality that could help bridge this gap.

## The utility of OMT: evidence review

The evidence base for OMT spans multiple populations relevant to palliative care, encompassing diverse age ranges and disease pathologies. The most robust data comes from patients with musculoskeletal disorders. In patients with obesity and low back pain, OMT improved range of motion, disability, and pain (3). Similarly, the addition of OMT to standard care for patients with fibromyalgia led to significant improvements in perceived pain, pain threshold, perceived functional ability, attitude toward treatment, and activities of daily living (4). A two-phase clinical intervention study in patients with late whiplash syndrome demonstrated a 37% reduction in neck pain and disability after a course of OMT, along with substantial improvements in physical and mental domains of quality of life and a 64% reduction in PTSD rates (5).

Beyond specific musculoskeletal conditions, OMT can improve both nociceptive and neuropathic acute and chronic pain in patients affected by a range of physical ailments (6). These effects may partially stem from anti-inflammatory mechanisms, including associations between IL-6, IL-1β, and reductions in TNF-α and substance P (7, 8). A comprehensive mapping review identified 146 studies documenting biological modifications following OMT across several body systems, with the strongest evidence in neurophysiological correlates and musculoskeletal changes, further supporting a mechanistic basis for the clinical effects observed (9). These immunomodulatory effects may also at least partly explain preliminary evidence suggesting a reduction in overall symptoms, improved efficiency of standard care, and decreased long-term consequences of COVID-19 infection in patients treated with OMT (10). Further research is needed to elucidate the mechanisms behind these benefits and to identify possible avenues for future investigation.

Given the prevalence of cancer among patients receiving palliative care, the application of OMT in oncology populations warrants particular attention. The American Society for Clinical Oncology (ASCO) 2024 Palliative Care Guideline Update references a model of “precision palliative care” that promotes a targeted and efficient use of resources and expertise to address multiple forms of distress—physical, psychosocial, spiritual—for patients with advanced cancer (11). The Society for Integrative Oncology (SIO) and ASCO jointly published a guideline on integrative medicine for pain management in oncology, which recommends massage for patients receiving palliative or hospice care based on moderate-quality evidence, while noting that research on other manual interventions—including OMT—remains limited in both quantity and quality (12). This evidence gap underscores the need for further investigation of OMT specifically within oncology and palliative populations.

Despite this gap, the existing evidence for OMT improving cancer-related pain is encouraging. A single-institution placebo-controlled randomized control trial (RCT) evaluating OMT for pain in a palliative care unit demonstrated a 43.2% reduction in pain scores and a 31.58% reduction in patient-controlled analgesia (PCA) doses over the course of treatment. These benefits were noticeable immediately after treatment and continued through the last day of evaluation. The results also demonstrate that OMT treatments were associated with increased quality of life scores (7). A small study of geriatric patients with cancer similarly showed that manipulative treatments improved pain and quality of life (13). A systematic review and meta-analysis evaluating manual therapy (massage, acupressure, OMT, and other physical therapies operated by manual touch) in patients with cancer found that, compared with standard treatment, manual therapy produced a significant improvement in pain scores (14). More recently, a 2026 systematic review of instrumental physical therapies and manual therapy in cancer patients found a positive effect of physiotherapy interventions on pain reduction in cancer survivors both during and after chemotherapy, with no absolute contraindications identified—including in patients with metastatic disease. The authors recommended implementing manual therapy as part of a multimodal approach rather than as an isolated intervention (15).

The role of OMT in managing non-pain symptoms in people with cancer remains less well studied, though the evidence to date is favorable. A 2019 RCT evaluated the impact of visceral OMT on nausea, vomiting, constipation, and overall quality of life in breast cancer patients receiving chemotherapy. Although the incidence of gastrointestinal symptoms did not change, the impact of these symptoms on overall quality of life improved significantly in patients treated with OMT (16). A feasibility study of OMT in children and adolescents with hematologic malignancies demonstrated improved limb range of motion and mobility of the spine, chest, and abdomen (6).

Precision medicine in palliative pain management is also increasingly incorporating pharmacogenomics, such as CYP2D6 testing, to guide opioid selection and dosing (17, 18). CYP2D6 is an important enzyme for the metabolism of opioids. Though the 2022 ASCO guideline for the use of opioids for cancer-related pain notes that “evidence remains insufficient to recommend for or against the use of genetic testing, such as for polymorphism of CYP2D6, to guide opioid dosing” (19), approximately 15% of the population has reduced or absent CYP2D6 activity, and an additional ~3% are ultrarapid metabolizers (20). As ongoing investigation clarifies the patient-specific utility of opioids for pain management, non-opioid modalities such as OMT become increasingly relevant as adjunctive or alternative approaches.

The benefits of OMT extend beyond the physical dimension. In addition to improving somatic dysfunction, OMT provides the opportunity for therapeutic physical touch—an intervention that confers benefits not only for physical health but also in the psychosocial and interpersonal realms, with reductions in pain, anxiety, and depression (21). A systematic review and meta-analysis of RCTs found that depression was significantly reduced in patients receiving OMT, particularly those receiving treatment for pain (22). A single-blinded RCT evaluating OMT for chronic neck pain demonstrated clinically meaningful improvements in depression scores, sleep, fatigue, and disability (23). A prospective observational study of patients receiving OMT for musculoskeletal disorders reported improvements in vitality, mental health, and social function in addition to physical functioning and pain scores after 4 weeks of treatment (24). These psychosocial dimensions were further illuminated by a qualitative analysis of concomitant OMT with standard cancer treatments in a palliative care unit, in which patients reported improvements in sleep, pain, and fatigue. Notably, patients attributed these benefits to “more than just a manual therapy,” describing the effects of therapeutic touch and likening treatments to a meditation session. Patients valued the non-pharmaceutical approach and the fact that the treating provider was a palliative care physician who understood the severity of their illness (25).

## Barriers to implementation

Despite these broad benefits, multiple barriers to implementation remain. Because OMT training distinguishes the education and practice of Doctors of Osteopathic Medicine (DO) from Medical Doctors (MD), OMT is not taught to MD physicians. Only about 10% of practicing Hospice and Palliative Care physicians are DOs (26), and not all DOs practice OMT, imposing a significant limitation on patient access. Further, over 50% of practicing DOs report using OMT on fewer than 5% of their patients (27). Discussions have emerged around DO physicians teaching their MD counterparts OMT basics (28, 29), though such initiatives remain limited in scope.

A 2025 qualitative study provides the most detailed characterization of these barriers to date. Through semi-structured interviews with 16 DOs at Mayo Clinic and Mayo Clinic Health System, the authors identified six overarching themes that hinder OMT integration: system, capability, utility, training, attitudes and beliefs, and resources. At the systemic level, patient panel management demands and limited practice autonomy constrain the ability of DOs to incorporate OMT into their workflows. Capability barriers include loss of technical skill over time from disuse, while utility concerns reflect uncertainty among some physicians about the clinical scenarios in which OMT adds value beyond standard care. Training-related barriers encompass both the variability of OMT education across osteopathic medical schools and the limited availability of continuing education opportunities. Attitudinal barriers include skepticism from non-DO colleagues and, in some cases, from DOs themselves regarding the evidence base for OMT. Finally, resource barriers—including time constraints, scheduling complexity, inadequate office space, and limited patient awareness—emerged as among the most significant obstacles. The authors concluded that addressing these barriers will require intentional support from institutional leadership to improve access to OMT (30).

These findings are consistent with broader patterns of underutilization of integrative modalities in oncology and palliative care. A 2025 global survey by the Multinational Association of Supportive Care in Cancer (MASCC) and the Society for Integrative Oncology found that 79% of respondents perceived integrative oncology modalities to be underutilized in cancer supportive care, with financial barriers and limited training availability identified as contributing factors (31). Disparities in access further compound these barriers: men, patients with low levels of education, and non-White and Hispanic patients are significantly less likely to have received OMT, highlighting the need for equity focused implementation strategies (27). In a study of OMT understanding in pediatric oncology, no participant—patients, caregivers, or oncology clinicians—had more than “some” knowledge of OMT, though after receiving brief education, 95% of participants were receptive to OMT for supportive care (32).

## Conclusion

OMT represents a personalized, non-pharmacologic intervention that aligns with the principles of precision medicine and addresses the multidimensional symptom burden encountered in palliative care. The existing evidence demonstrates benefits across musculoskeletal pain, cancer-related pain, and psychosocial well-being, with emerging data supporting its role in managing non-pain symptoms such as nausea, fatigue, and sleep disturbance. The individualized nature of the structural examination and treatment plan—tailored to each patient’s unique somatic dysfunction—embodies the foundational concept of precision palliative care.

However, significant barriers to widespread implementation remain, including the limited number of DO-trained palliative care physicians who practice OMT, time constraints within clinical workflows, and low patient and provider awareness of OMT as a therapeutic option. Addressing these barriers will require interprofessional education initiatives, institutional support for OMT integration, and continued research to strengthen the evidence base.

Future investigations should prioritize well-powered, multi-center RCTs evaluating OMT for both pain and non-pain symptoms in palliative populations. Studies examining the immunomodulatory mechanisms underlying OMT’s clinical effects, as well as the intersection of pharmacogenomics-guided opioid management with non-pharmacologic modalities like OMT, represent promising avenues for advancing precision palliative care. As the field moves toward more targeted and efficient models of symptom management, OMT offers a patient-centered approach that complements pharmacologic strategies and honors the holistic goals of palliative medicine.

## Data Availability

The original contributions presented in the study are included in the article/supplementary material, further inquiries can be directed to the corresponding author.

## Author disclaimer

This research was supported [in part] by the Intramural Research Program of the National Institutes of Health (NIH). The contributions of the NIH author(s) are considered Works of the United States Government. The findings and conclusions presented in this paper are those of the author(s) and do not necessarily reflect the views of the NIH or the U.S. Department of Health and Human Services.
